# Antioxidant and Neuroprotective Properties of *Eugenia dysenterica* Leaves

**DOI:** 10.1155/2018/3250908

**Published:** 2018-09-19

**Authors:** Douglas Vieira Thomaz, Luanna Fernandes Peixoto, Thiago Sardinha de Oliveira, James Oluwagbamigbe Fajemiroye, Hiasmin Franciely da Silva Neri, Carlos Henrique Xavier, Elson Alves Costa, Fernanda Cristina Alcantara dos Santos, Eric de Souza Gil, Paulo César Ghedini

**Affiliations:** ^1^Instituto de Ciências Biológicas, Universidade Federal de Goiás, Goiânia, GO, Brazil; ^2^Faculdade de Farmácia, Universidade Federal de Goiás, Goiânia, GO, Brazil

## Abstract

*Eugenia dysenterica* ex DC Mart. (Myrtaceae), popularly known as “cagaita,” is a Brazilian plant rich in polyphenols and other antioxidant compounds. Aiming to evaluate the potential use of cagaita in pathologies involving oxidative stress, such as neurodegenerative disorders, this study investigated its antioxidant potential and neuroprotective effect. Electrochemical approaches and aluminium-induced neurotoxicity were used to determine respectively *in vitro* and *in vivo* antioxidant properties of cagaita. Voltammetric experiments were carried out in a three-electrode system, whose working electrode consisted of glassy carbon. Male Swiss mice were administered with AlCl_3_ orally at a dose of 100 mg/kg/day and with cagaita leaf hydroalcoholic extract (CHE) at doses of 10, 100, and 300 mg/kg/day. The redox behavior of CHE presented similar features to that of quercetin, a widely known antioxidant standard. CHE prevented mouse memory impairment which resulted from aluminium intake. In addition, biochemical markers of oxidative stress (catalase, superoxide dismutase activity, and lipid peroxidation) were normalized by CHE treatment. The potential of CHE to prevent aluminium-induced neurotoxicity was reflected at the microscopic level, through the decrease of the number of eosinophilic necrosis phenotypes seen in treated groups. Moreover, the protective effect of CHE was similar to that of quercetin, which was taken as the standard. These findings showed that the CHE of cagaita leaves has a potential to protect the brain against oxidative-induced brain damage.

## 1. Introduction

Oxidative stress is known to promote a myriad of pathological conditions due to prooxidant compound accumulation. Regarding lipid-rich organs such as the brain, reactive oxygen species (ROS) may lead to lipid peroxidation, thus impairing neurotransmitter signaling, which further worsens the neuronal functions [1, 2]. Brain cells are particularly sensitive to oxidative damage due to their high oxygen uptake; therefore, the regular consumption of antioxidants is important concerning neuroprotection [3, 4].

Although brain tissue contains endogenous antioxidants capable of ROS reduction, some enzymes also contribute to promote redox homeostasis. Nevertheless, the hindrance of this antioxidant system culminates in oxidative-related neurotoxicity [3, 4]. Moreover, exogenous factors such as heavy metals and aluminium poisoning may exert prooxidative behavior as well as promote steric hindrance of antioxidant enzyme active sites [5, 6].

Herbal mater is regarded as the major antioxidant source in medicine due to the high reducing power of many plant secondary metabolites. Amongst these compounds, shikimate pathway byproducts such as polyphenols are heavily sought after due to their reversible redox behavior. Although ubiquitarily distributed in vegetal kingdom, high concentration of these products can be found in species such as “cagaita” (*Eugenia dysenterica* ex DC Mart.—Myrtaceae), whose phenolic content is therapeutically employed in Brazilian folk medicine for a variety of uses, ranging from anti-inflammatory to laxative [7, 8].

Literature reports that polyphenolic-rich cagaita extracts improve glucose homeostasis of obese mice by attenuating hepatic gluconeogenesis and inflammation, as well as exhibit cytotoxic effect against SH-SY5Y human neuroblastoma cell line [8]. Cagaita's main phytocompounds, quercetin and catechin, are known for their beneficial action in human health due to anticancer, neuroprotective, and anti-inflammatory activities. Moreover, these phytochemical markers allow sample redox reversibility through catechol/quinone conversion [8] and therefore further strengthen antioxidant activity [8, 9].

Amongst assessment tools capable of antioxidant evaluation, methods such as voltammetry may provide increased sensibility and selectivity when compared to colorimetric tests [10] and can be furthermore used to electrochemically characterize a complex sample such as vegetal mater. Through such methods is possible to understand the redox features of a pool of compounds and provide important information about the thermodynamical feasibility of ROS reduction [11, 12].

In view of cagaita's applicabilities concerning neuroprotection and the importance of redox process characterization under the light of antioxidant activity assessment, this work is intended to provide a broad study regarding the neuroprotective and antioxidant features of the crude hydroalcoholic extract of cagaita leaves (CHE).

## 2. Material and Methods

### 2.1. Animals

Male Swiss mice (25–30 g) from the colony of the Federal University of Goiás were used in this study. The animals were housed under a controlled 12 h light/dark cycle and stable temperature (22–23°C) with free access to food and water. All experiments were conducted in accordance with the Sociedade Brasileira de Ciência em Animais de Laboratório (SBCAL) and were approved by the local Ethics in Research Committee (Protocol CEUA/UFG 53/2016).

### 2.2. Materials and Reagents

Bovine serum albumin, quercetin, aluminium chloride, 5:5-dithiobis-2-nitrobenzoate, acetylcholine, and epinephrine bitartrate were purchased from Sigma. Trichloroacetic acid (TCA) and glycine were purchased from Vetec. The compounds n-butanol and Na_2_HPO_4_ were purchased from Synth.

Furthermore, the present work employed the reagents NaH_2_PO_4_H_2_O (Cromoline), hydrogen peroxide (CRQ), thiobarbituric acid (TBA) (TediaBrasil), and coomassie brilliant blue (Amresco). All electrolyte salts, solvents, and reagents were of analytical grade. Electrolyte solutions were prepared with double distilled Milli-Q water (conductivity ≤ 0.1 *μ*S cm^−1^) (Millipore S. A., Molsheim, France).

### 2.3. CHE Preparation

Cagaita leaves were collected in Trindade, Goiás, Brazil, after identification. The vegetal mater was thereafter dried, milled, and stored at −20°C. A sample of cagaita was deposited at the Federal University of Goiás (UFG) herbarium under the code 50091.

The extract was prepared focusing on polyphenol extraction; henceforth, 50 g of cagaita vegetal material was suspended in a mix of 0.7 L of ethanol p.a. and 0.3 L of distilled water. The prepared material was macerated for 48 hours and then filtered.

The filtered fluid was reused (*n* = 3) to maximize extraction. The extract was thereafter concentrated in rotary evaporator and lyophilized. Dried CHE extract was stored in amber flasks at 4°C prior analysis.

### 2.4. Electrochemical Assays

Voltammetric experiments were carried out in a potentiostat/galvanostat Autolab III® integrated to the GPES 4.9® software, Eco-Chemie, Utrecht, Netherlands. The measurements were performed in a 1.0 mL one-compartment/three-electrode system electrochemical cell consisting of glassy carbon electrode (GCE) 1.0 mm^2^ area, a Pt wire, and Ag/AgCl/KCl_sat_ electrode (Lab Solutions, São Paulo, Brazil), representing the working electrode, the counter electrode, and the reference electrode, respectively.

Experimental conditions for square wave voltammetry (SWV) were as follows: scan rate from 0 to 1 V; pulse amplitude 50 mV; frequency (f) 50 Hz; and a potential increment of 2 mV, corresponding to an effective scan rate (*υ*) of 100 mV s^−1^. The experimental conditions for differential pulse voltammetry (DPV) were pulse amplitude 50 mV, pulse width 0.5 s, and scan rate 10 mV s^−1^. All voltammetric assays were performed in 0.1 M phosphate-buffered solution (PBS) pH 7.0.

All experiments were performed in triplicates, DPV results were background-subtracted and baseline-corrected to provide better data visualization, and all data was analyzed and treated with Origin 8® software.

### 2.5. Experimental Design

Animals were segregated in 6 groups (I to VI) (*n* = 10 each group) and undergone chronic treatment for 90 days. Treatment solutions were administered through gavage (0.1 mL/10 g). Treatment I was designed as a control group (vehicle-distilled water); therefore, only water was administered, while treatments II to VI were test groups. Henceforth, AlCl_3_ solution (100 mg/kg) was administered, on the morning, from day 0 to day 90. After the 45^th^ day, a second treatment was orally administered in the afternoon. The second treatment consisted of distilled water (groups I and II), quercetin 30 mg/kg (III), CHE 10 mg/kg (IV), CHE 100 mg/kg (V), and CHE 300 mg/kg (VI). The CHE concentrations herein used were selected according to an experimental optimization conducted by our research group (data not shown).

After treatment period, behavior was evaluated (memory and locomotor activity) and then the animals were sacrificed by cervical dislocation and the brain cortex and hippocampi were removed and stored at 4°C for biochemical and histopathological assays.

### 2.6. Behavioral Studies

In order to assess the neuroprotective properties of CHE against aluminium-induced neurotoxicity, three behavioral tests were conducted, namely, step-down test to evaluate short- and long-term memories [13], open-field, and chimney tests to evaluate locomotor activity [14, 15].

### 2.7. Step-Down Test

Step-down apparatus was a box (30 cm × 20 cm × 20 cm high) with three walls of stainless-steel and one wall of Plexiglas, featuring a grid floor (made up of 3 mm stainless-steel rods set 1 cm apart) and one platform (8.5 cm × 1.5 cm × 20 cm high) placed in one side of the apparatus, which was connected to a shock generator and a scrambler (Insight Ltda., Ribeirão Preto, SP, Brasil).

Mice were gently placed on the platform upon stepping four paws onto the grid floor when they received a 0.2 mA/s shock. The latency of the step-down motion was recorded, and the training consisted of one trial. During the retention test session, mice were placed on the platform, but no shock was given when they stepped down. The latency of the step-down motion onto the grid was also recorded in the test session. To evaluate the short- and long-term memory, animals had their test session at 90 min and 24 h, respectively. The box was illuminated with a 15 W light bulb during the experimental period. Retention test scores were expressed as test *minus* training step-down latency. Passive avoidance behavior based on negative reinforcement was used to examine the short- and long-term memory [13].

### 2.8. Open-Field Test

Mice were tested for their spontaneous locomotor activity and exploratory behavior in a transparent Plexiglas arena (30 × 30 × 15 cm) with a black Plexiglas floor divided in 9 equal squares. Animals were assessed on the number of squares crossed in a 5 min period [14].

### 2.9. Chimney Test

The chimney test consisted of evaluating the inability of animals to climb backward up through a Plexiglas tube (3 cm, inner diameter × 30 cm, length) within 30 s. The results were presented as time(s) to climb backwards out of the tube [15].

### 2.10. Biochemical Assays

Twenty-four hours after the last behavioral test, the animals were anesthetized with isoflurane. Subsequently, mice were euthanized by blood extraction through cardiac puncture and the cerebral tissue was removed. Animal's cerebral cortices and hippocampi were immersed in phosphate-buffered solution pH 7.4 at a proportion of 1 : 5 *w*/*v* and homogenized in a tissue homogenizer (Homo Mix). The resulting colloid was centrifuged at 4000 rpm for 20 minutes at 4°C, and the supernatant (biological sample) was assessed on its protein content by the Bradford method [16]. Thereafter, the supernatant was also used to assess thiobarbituric acid-reactive species (TBARS), total superoxide dismutase activity, and catalase activity.

### 2.11. Determination of TBARS Levels

This estimation was done by following the method mentioned by Ohkawa et al. [17]. It is based on the reactivity of an end product of lipid peroxidation, malondialdehyde (MDA) with thiobarbituric acid (TBA) to produce a red adduct. The samples (tissue supernatant) were incubated at 100°C for 60 min in acid medium and, thereafter, were centrifuged 5000 × *g* for 5 min, and the reaction product was determined at 532 nm. The level of lipid peroxides was expressed as nmoles of MDA released/mg protein.

### 2.12. Superoxide Dismutase (SOD) Activity

The principle of this method is the ability of superoxide dismutase enzyme to inhibit the autoxidation of epinephrine. The cerebral cortex and hippocampus supernatants were incubated with epinephrine bitartrate 60 mM, and the sample color intensity was measured at 480 nm, according to the method of Misra and Fridovich [18]. Unit/*μ*L activity was determined and calculated the total SOD activity as unit enzyme activity per mg protein.

### 2.13. Catalase Activity

Catalase catalyzes the decomposition of H_2_O_2_ to give H_2_O and O_2_. The activity of catalase enzyme can be measured by following either the decomposition of H_2_O_2_ or the liberation of O_2_. The rate of decomposition of H_2_O_2_ of the cerebral cortex and hippocampus samples was spectrophotometrically measured from changes in absorbance at 240 nm. The catalase activity was expressed as nmol/mg protein, according to the method described by Aebi [19].

### 2.14. Morphometric and Histopathological Analysis

Animal's hippocampi were fixed in methanol/chloroform/acetic acid solution (6 : 3 : 1) and then dehydrated in a crescent concentration of ethanol. The dehydrated material was clarified with xylol and embedded in Paraplast (Histosec, Merck). After inclusion, the material was sectioned at 5 *μ*m and stained by a hematoxylin-eosin method. Morphometry was carried out by analyzing CA1 hippocampus pyramidal layer thickness (*μ*m; 60 measures/group). Relative frequencies (%) of viable neurons and necrotic eosinophilic neurons of the CA1 layer were performed using Weibel's multipurpose graticulate with 130 points and 10 test lines [20]. Thirty microscopic fields were chosen at random from each experimental group (six fields per animal; *n* = 5). The relative values were determined by counting the coincident points in the test grid and dividing them by the total number of points. The hippocampal sections were analyzed using an Olympus BX43 light microscope (Olympus, Japan). All analyses were conducted using Image Pro-Plus program version 6.1 (Media Cybernetics Inc., Silver Spring, MD, USA). Values were presented as arithmetic mean ± standard error of the mean.

### 2.15. Statistical Analysis

Data was subjected to Student's *t*-test for two group comparisons, two or more groups were analyzed, and analysis of variance was employed using Tukey-Kramer's test. Statistical significance was considered to *p* < 0.05. All data was processed using GraphPad Prism software (GraphPad version 6.00, San Diego, CA).

## 3. Results and Discussion

### 3.1. Electrochemical Assays

**Figure 1:**
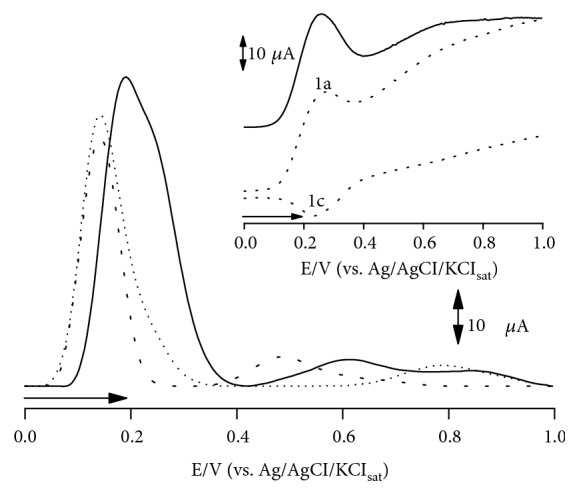
DP voltammogram of CHE (▬), quercetin (▪▪▪), and catechin (•••). Pulse amplitude 50 mV, pulse width 0.5 s, and *υ* of 10 mV s^−1^. Insert: SW voltammograms of CHE. CHE anodic peak (1a) and cathodic peak (1c) herein highlighted pulse amplitude 50 mV, frequency of 50 Hz, and potential increment of 2 mV, corresponding to an effective *υ* of 100 mV s^−1^. Analysis carried out in 0.1 M PBS, pH 7.0.

The redox behavior of CHE was evaluated by means of DPV and SWV (Figure 1).

Figure 1 shows the evidence an anodic peak, 1a, at ca. 0.2 V, which is correlated to the oxidation of electroactive species present in the sample. This peak potential value is akin to the observed for catechol moiety in natural antioxidants, such as catechin and quercetin. This fact is corroborated by literature reports concerning ED leaf extract, which is described as high polyphenolic content [11].

The antioxidant activity can only be deemed to assist neuroprotection when ROS reduction is thermodynamically feasible. Henceforth, CHE presents outstanding antioxidant quality, as the anodic peak occurred at 0.2 V, wherein higher reducing power at physiological pH is associated to peak potentials below 0.5 V [8, 9].

Furthermore, SWV allowed the visualization of a cathodic peak 1c, which is intimately correlated to anodic peak 1a. These peaks configure reversibility, as their anodic and cathodic current ratio (*I*_pa_/*I*_pc_) was quite close to 1. This pattern implies that sample compounds may repeatedly undergo oxidation to restore endogenous antioxidants in physiological environment. Henceforth, the thermodynamic reducing feasibility and their reversible profile imply that CHE possesses noteworthy antioxidant properties [8, 9].

### 3.2. Behavioral Studies

**Figure 2:**
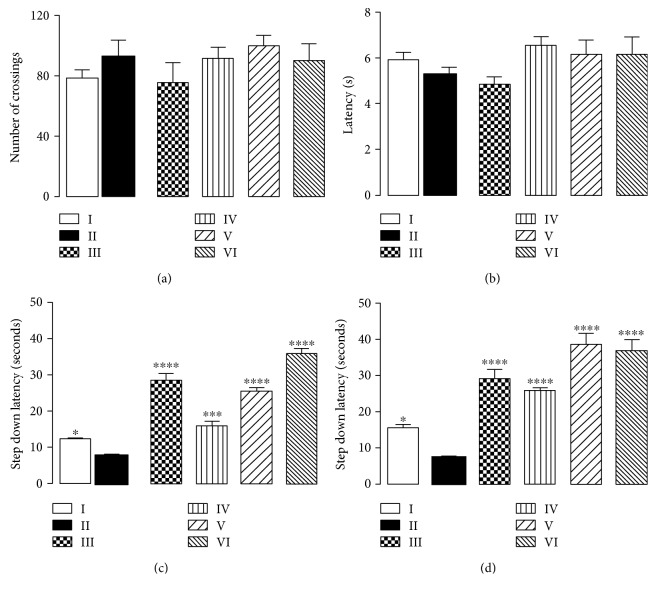
Effect of CHE treatment on locomotor activity (a and b) and memory (c and d) of mice subjected to 90 days of aluminium exposure. (a) Number of crossings of mouse groups as evaluated in the open-field test. (b) Time(s) to climb backwards out of the tube within 30 sec of the examined animals in the chimney test. (c and d) Latencies of retention time(s) in mice as evaluated in the step-down test at 90 min (c) and 24 h (d) after shock challenge, respectively. Each column represents mean ± SEM of 10 animals. ^∗^*P* < 0.05, ^∗∗∗^*P* < 0.001, and ^∗∗∗∗^*P* < 0.0001 in comparison to group II. (I) Control group; (II) aluminium group; (III) quercetin 30 mg/kg; (IV) CHE 10 mg/kg; (V) CHE 100 mg/kg; and (VI) CHE 300 mg/kg.

Behavioral tests gather important data concerning the extent of neurological damage. Aspects such as short- and long-term memories are assayed in order to study memory retention capabilities, whereas locomotor, exploratory activity, and motor impairment are assayed to study the influence of the tested compounds in motor system. These aspects were herein assessed respectively through step-down, open-field, and chimney tests. Results are displayed in Figure 2.

Figure 2 exhibits step-down test results and shows that during training, all groups presented similar profiles, whereas for short-term memory (STM) and long-term memory (LTM), group II presented the lowest memory retention. Since this group was solely treated with AlCl_3_, it can be attested that this metal promotes memory impairment. Nonetheless, the neurotoxicity model herein employed is extensively used in research to mimic neurodegeneration and is considered optimal to simulate Alzheimer's disease features [5, 6].

The quercetin-treated group (III) exhibited higher memory retention than the control group for both STM and LTM, which is justified by this compound's neuroprotective properties. As all polyphenols, quercetin promotes ROS scavenging, which minimizes oxidative stress and oxygen-related injuries in brain mater [12–21].

Animals treated with CHE exhibited crescent memory retention according to the rise in concentration. Group VI, which was treated with the highest extract concentration (300 mg/kg), exhibited the best STM retention, even surpassing the quercetin-treated group (III) (Figure 2). Moreover, it was seen that LTM retention reaches a plateau at a concentration of 100 mg/kg, and further concentrations did not exhibit any enhancement of this feature. Results imply henceforth that CHE does indeed promote memory enhancement, and this propriety is seemingly concentration dependent up to 100 mg/kg and 300 mg/kg for LTM and STM, respectively. The findings herein obtained are justified by cagaita antioxidant activity, which promotes neuroprotection [11]. As seen in voltammetrical assessment, endogenous antioxidant renewal by CHE is thermodynamically feasible, which implies that ROS are efficiently scavenged.

Open-field and chimney test results evidenced that the extracts did not impair locomotion. A noteworthy finding is that even at high concentration (300 mg/kg), the extract did not promote any statistically significant interference on motor system. Results imply therefore that CHE promotes memory retention without impairing motor skills.

### 3.3. Biological Sample Antioxidant Analysis

**Figure 3:**
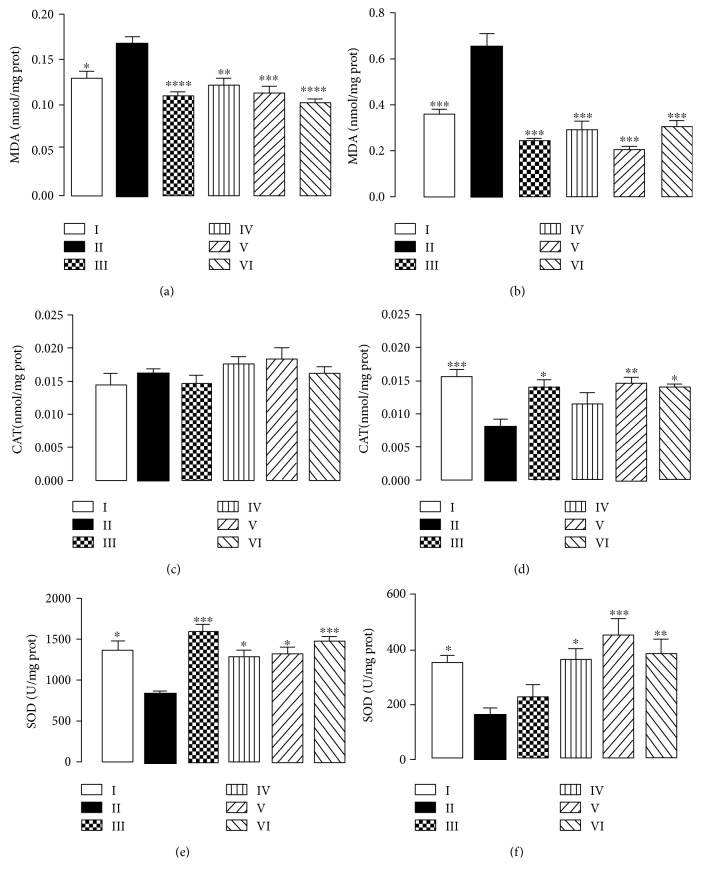
Effect of CHE treatment on malondialdehyde (MDA) concentration in animal cortices (a) and hippocampi (b), catalase (CAT) activity in animal cortices (c) and hippocampi (d), and superoxide dismutase (SOD) activity in animal cortices (e) and hippocampi (f). All results evaluated from mice subjected to 90 days of aluminium exposure. Each column represents mean ± SEM of 10 animals. ^∗^*P* < 0.05, ^∗∗∗^*P* < 0.001, and ^∗∗∗∗^*P* < 0.0001 in comparison to group II. (I) Control group; (II) aluminium group; (III) quercetin 30 mg/kg; (IV) CHE 10 mg/kg; (V) CHE 100 mg/kg; and (VI) CHE 300 mg/kg.

In order to complement electrochemical assessment on antioxidant quality, CHE had its antioxidant activity analyzed by assessing cortex and hippocampus response towards TBARS, superoxide dismutase, and catalase activity. Results are displayed in Figure 3.

Figure 3 displays cortex and hippocampus response to TBARS. Malondialdehyde is a lipid peroxidation product whose increased concentrations imply elevated oxidative stress, and this compound is moreover highly reactive towards thiobarbituric acid. The aluminium-treated group (II) presented a rise of malondialdehyde concentration in both cortices and hippocampi of treated animals, whereas all other treatment groups did not display statistically significant variation compared to control (I) response. Results evidence that AlCl_3_ does promote lipid peroxidation, which is in accordance to literature. Although not a direct prooxidant, aluminium potentiates iron-mediated ROS synthesis in Fenton reaction and promotes steric hindrance of antioxidant enzyme active sites [5, 6]. CHE as well as quercetin exhibited similar results to that of the control group, which implies that ROS were unable to promote lipid peroxidation in these samples.

Concerning total superoxide dismutase activity in cortices and hippocampi, this enzyme is known to be hindered by aluminium salts, and results display the same trend, as group II presented statistically significant activity reduction. Although all other treated groups presented similar features to control concerning animal cortices, hippocampus response to quercetin implied that this compound hindered enzyme activity, whereas CHE increased its activity. Literature reports that polyphenols may interact with proteins depending on their chemical structure and either promote or hinder their activity [22]. Results imply therefore that CHE constituents do promote superoxide dismutase activity.

Literature reports that chronic aluminium poisoning leads to the decrease of manganese-dependent SOD activity (mitochondrial SOD) in mouse brain. Moreover, exposure to this metal ultimately decreases Lon protease mRNA expression. Since this enzyme is involved in the removal of oxidized proteins from mitochondria, the decline of its expression may enhance the damage promoted by ROS. Although the present work did not evaluate MnSOD, the overall decline of total SOD (manganese-, copper-, and zinc dependent isoforms) in group II implies the detrimental effects of aluminium exposure, as well as indicates the SOD-promoting effects of CHE [23, 24].

Although we opted in the present work to provide a biochemical approach of cagaita's leaf antioxidant mechanisms, literature reports that the aluminium neurotoxicity model herein employed does promote memory impairment through transcriptional regulation as well as impacting multiple signal transduction pathways. The chronic exposure to aluminium diminishes early- and late-phase long-term potentiation, as well as decreases hippocampal cAMP, cPKA, pCREB, BDNF, and c-jun. Since all these biomolecules are involved in memory retention, CHE may possibly exert its neuroprotective properties through mechanisms other than plain antioxidant action; however, more studies are necessary to investigate it further [24, 25].

Regarding catalase activity in animal's cortices and hippocampi, this enzyme is one of the main components in an organism's ROS-reductive arsenal. Results displayed that catalase activity was slightly enhanced by CHE in the cortices, whereas enzymatic activity rose significantly in the hippocampi of the treated animals. Results once again demonstrate that CHE potentiates ROS scavenging by promotion of antioxidant catalytic system, which sheds more light on this sample's antioxidant proprieties.

### 3.4. Morphoquantitative Stereological Analysis

**Figure 4:**
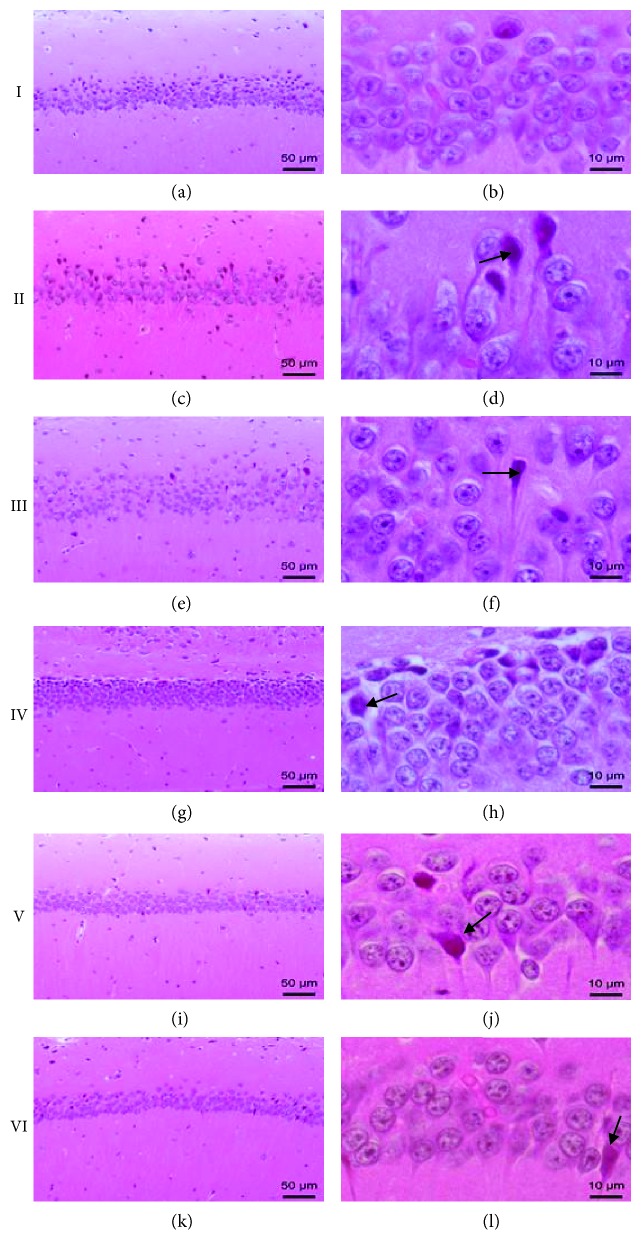
Histopathological features of CA1 region of the hippocampus. Control mouse brain section (I) showed normal histological structure of the neurons in the hippocampus. All intoxicated mice showed nuclear pyknosis and degeneration in the neurons. Treatment of quercetin 30 mg/kg (III), CHE 10 mg/kg (IV), CHE 100 mg/kg (V), and CHE 300 mg/kg (VI) showed nuclear pyknosis and degeneration in few neurons of the CA1 area of the hippocampus. H&E. Scale bar: 50 *μ*m in (a, c, e, g, i, and k) and 10 *μ*m in (b, d, f, h, j, and l). (I) Control group; (II) aluminium group; (III) quercetin 30 mg/kg; (IV) CHE 10 mg/kg; (V) CHE 100 mg/kg; and (VI) CHE 300 mg/kg.

Morphoquantitative and stereological data evidenced that treatments deeply alter CA1 hippocampus pyramidal cell layer architecture and thickness, as shown in Figure 4.

Results showed that in the control group, pyramidal cell layer thickness was of 55.0 ± 0.8 *μ*m and regularly distributed. Cells contained a single nucleus, up to three nucleoli, and basophilic cytoplasm (Figure 4).

**Figure 5:**
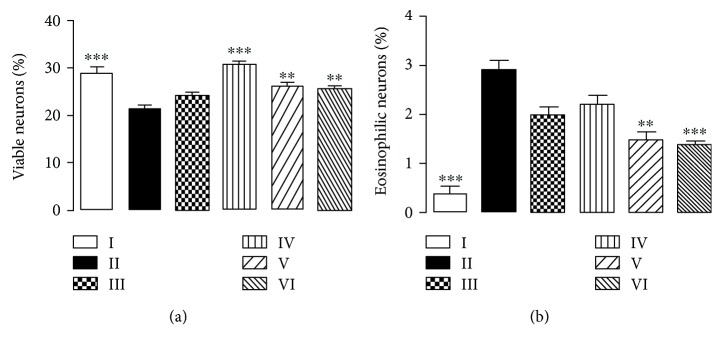
Relative frequency (%) of viable neurons (a) and necrotic neurons (b) in the CA1 area of the hippocampus. Each column represents mean ± SEM of 10 animals. ^∗∗^*P* < 0.001 and ^∗∗∗^*P* < 0.0001 in comparison to group II. (I) Control group; (II) aluminium group; (III) quercetin 30 mg/kg; (IV) CHE 10 mg/kg; (V) CHE 100 mg/kg; and (VI) CHE 300 mg/kg.

AlCl_3_ exposure expressively reduced CA1 layer (40.3 ± 0.9 *μ*m), and cells became dispersed. Both tissue architecture and cells were altered by aluminium exposure, as cell cytoplasm increased in volume and presented more vacuoles than the untreated group. Aluminium treatment caused eosinophilic necrosis, reducing neuronal viability in the CA1 hippocampus layer (Figure 5).

The quercetin-treated group also presented CA1 layer width reduction (47.8 ± 1.3 *μ*m), albeit not as expressive as in the aluminium-treated group (II) (Figure 4). Cells presented themselves dispersed, and the occurrence of eosinophilic necrosis and viable neurons did not change when compared to the AlCl_3_-treated group (Figure 5).

In all CHE treatment groups, CA1 layer conserved architecture akin to the control group, albeit CHE concentrations of 10 mg/kg and 100 mg/kg (groups IV and V, respectively) still presented a slight thickness loss (48.9 ± 0.6 *μ*m and 48.5 ± 0.9 *μ*m, respectively). CHE's highest concentration of 300 mg/kg (VI) conserved the same thickness as the control group (56.8 ± 1.3 *μ*m). Viable neurons were more frequent in the CHE's lowest concentration (Figure 5). Although some eosinophilic necrosis phenotypes could still be seen, their occurrence was far less frequent than that in the AlCl_3_-treated group, especially in groups V and VI (Figure 5). Thus, morphological data indicate that neuron viability is best preserved at the CHE concentration of 10 mg/kg. However, CHE concentrations of 100 mg/kg and 300 mg/kg appear to be more effective in preventing neuronal death due to eosinophilic necrosis after 90 days of AlCl_3_ intake. As plant crude extracts contain a myriad of components [26, 27], we hypothesize that compounds in CHE do not act independently, presenting either synergistic or antagonistic effects that may explain the absence of a correlation between CHE doses and morphological tests. Moreover, this same interpretation may explain the results, in which CHE exhibited better effects than quercetin. However, further experiments need to be done to elucidate our interpretation. Henceforth, when the results herein depicted are analyzed under the light of CHE inherent antioxidant activity and its ROS scavenging enzyme promotion, our findings provide definitive evidence of the neuroprotective properties of this complex sample.

## 4. Conclusions

CHE constituents are able to efficiently scavenge ROS, and this process is thermodynamically feasible. Concerning behavioral assessment, CHE minimized memory loss without impairing animal movement. Biochemical analysis also proved that the extract herein used promotes superoxide dismutase and catalase activities, while simultaneously preventing lipid peroxidation. These findings were histologically assessed and proven, which sheds light on cagaita as a noteworthy source of neuroprotective compounds.

## Data Availability

All data used to support the findings of this study are included within the article.
